# Analysis of the unplanned reoperation following surgical treatment of pulmonary tumor

**DOI:** 10.1186/s13019-022-02064-6

**Published:** 2022-12-12

**Authors:** Long-Yong Mei, Yong-Geng Feng, Shao-Lin Tao, Bin Jiang, Fu-Qiang Dai, Jing-Hai Zhou, Cheng Shen, Wei Guo, Qun-You Tan, Bo Deng

**Affiliations:** Thoracic Surgery Department, Institute of Surgery Research, Daping Hospital, Army Medical University, Changjiang Branch St,10#, Yuzhong District, Chongqing City, 400042 China

**Keywords:** Thoracic surgery, Complications, Reoperation, Pulmonary tumor

## Abstract

**Background:**

In this study, we aimed to summarize the extremely important lesson and experience in the whole process of surgical treatments of lung tumors for the benefit of steps taken to prevent against unplanned reoperation.

**Methods:**

Demographical and clinical information of 7732 patients were retrospectively retrieved and reviewed, who were diagnosed with pulmonary tumor and underwent surgical treatments from January 2016 to March 2021. Those patients who underwent unplanned reoperation for the treatment of severe complications were focused carefully and analyzed meticulously.

**Results:**

A total of forty-one patients (41/7732) received 44 unplanned reoperations. Among them, eight and thirty-three patients were diagnosed with benign and malignant tumor, respectively. The incidence of unplanned reoperations seemed to be similar on both sides (Left vs. Right: 12/3231 vs. 29/4501, *p* = 0.103). Lobectomy plus segmentectomy is prone to reoperation (2/16, 12.5%) as compared to the other types of surgery. The complications leading to reoperation was hemothorax, including active hemorrhage (23/44, 52.3%) and clotted hemothorax (6/44, 13.6%), chylothorax (8/44, 18.2%), and the others (7/44, 15.9%) including bronchopleural fistula, torsion, or injury of right middle bronchus and pulmonary bulla rupture. The morbidity and mortality after unplanned reoperation were 17.1% (7/41) and 12.2% (5/41), respectively.

**Conclusions:**

Bronchi or vessel stumps, the surgical edges of the lung parenchyma, and pleural adhesions should be checked to avoid postoperative bleeding. Prophylactic ligation of the thoracic duct should be recommended in case of the suspected oily-like exudation in the lymph node bed. Smooth expansion of the middle lobe is important to avoid narrowing and torsion before transection of the bronchus.

## Background

In recent decades, minimally invasive thoracic surgery become common worldwide with the development of surgical mechanical device and accumulation of surgical skills [1]. As compared to thoracotomy, Video-Assisted thoracoscopic surgery (VATS) has fewer postoperative complications associated with less unplanned reoperation to treat that untoward complications [2, 3]. For instance, the incidence of overall complications following VATS lobectomy and segmentectomy for lung tumor treatment was reported to be 14% to 18%, while the 30-day perioperative mortality ranged from 0.9 to 1.4% [4, 5].

However, some major postoperative complications still need unplanned surgical treatment, which increases hospital stay and medical costs, and even more intractable complications and higher mortality rate due to second injury and anesthesia [6]. The incidence of unplanned reoperation after VATS lobectomy was 0.27–3.5% [7, 8], with the 90-day death 1.6% [8].

To our knowledge, the published study of unplanned reoperation is very rare although it is an important indicator of the quality of surgery. Therefore, in this study, we retrospectively studied and reviewed the cases with unplanned reoperation following lung-related tumor resection in the last five years, and aimed to summarize the extremely important lessons in the whole process of surgical treatments for for the benefit of steps taken to prevent against unplanned reoperation.

## Methods

### Patients

We retrospectively reviewed the medical records of the patients who were diagnosed with pulmonary tumor and underwent surgical treatments at the Thoracic Surgery Department, Daping Hospital, Army Medical University (Chongqing, China) from January 2016 to March 2021. We carefully retrieved and reviewed the detailed demographical, clinical, and pathological data of those patients who underwent unplanned reoperation for the treatment of complications. This retrospective study was approved by the Ethics Committee of Daping Hospital of Army Medical University NO.209 (2021) which waived the requirement for informed consent and was conducted in accordance with the Declaration of Helsinki and Ethical Guidelines for Medical and Health Research Involving Human Subjects.

Inclusion criteria are following: (i) Pulmonary lesions can be confirmed by Chest CT or PET-CT; (ii) The initial operations included exploratory thoracotomy, pneumonectomy, lobectomy, segmentectomy, and/or wedge resection; (iii) The approaches of initial operation included VATS, Robot-assisted thoracic surgery (RATS) or thoracotomy. (iv) The unplanned reoperation in the perioperative period was performed to treat complications due to the initial operation within 30-day.

Exclusion criteria are following: (i) Pulmonary sequestration, mediastinal tumor, and bronchiectasis were excluded by postoperative pathology; (ii) The unplanned reoperation was performed due to other reasons, rather than the complications caused by the first surgery.

### Statistical analysis

The statistical analysis was performed with SPSS v.26. The continuous data described as mean ± SDs were compared with Student's t-test. The categorical data were described as the ratio, which were compared with Pearson's χ2 or Fisher's exact test. The statistical significance was set at *p* < 0.05.

## Results

**Fig. 1:**
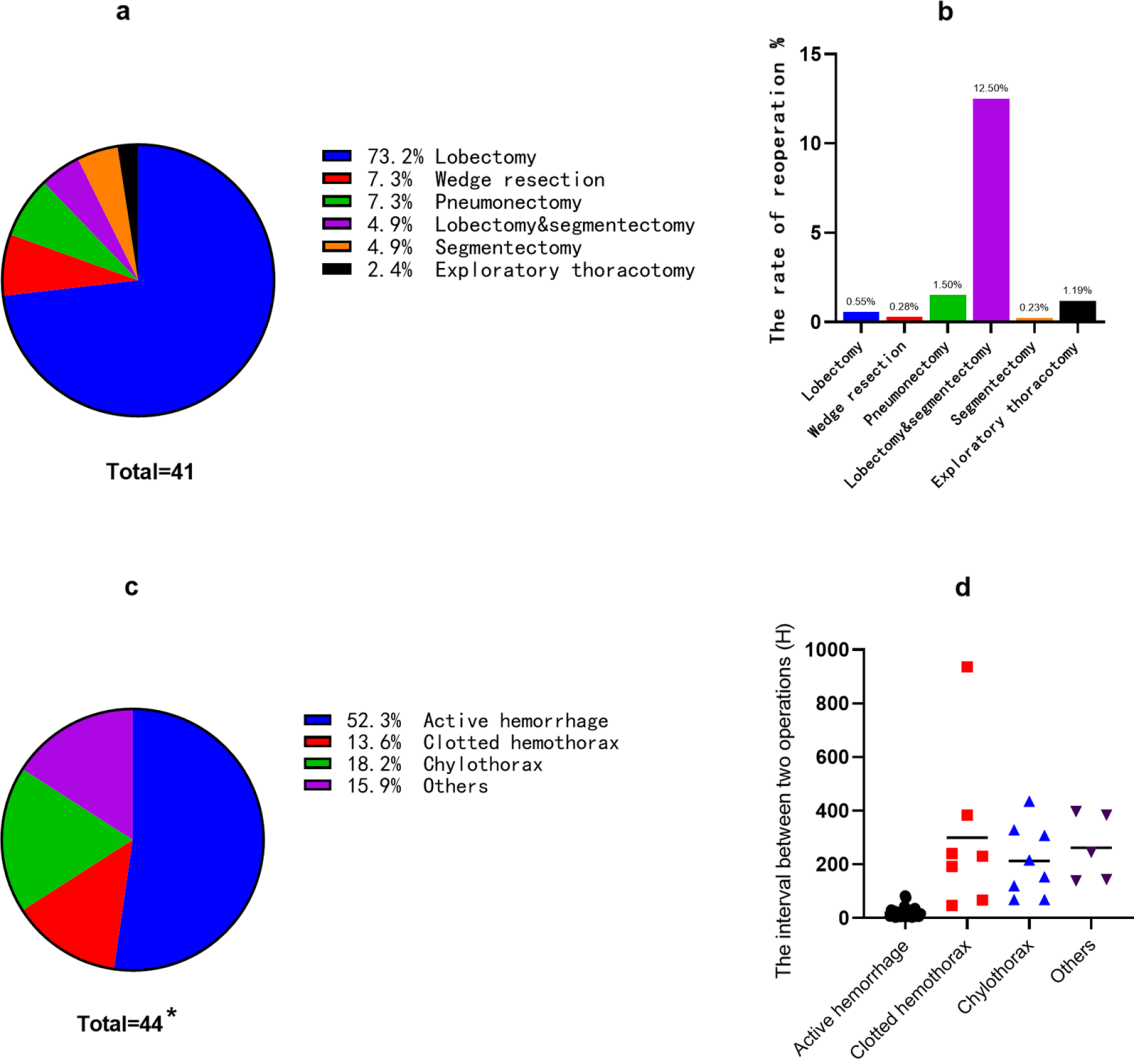
The clinical characteristics of the unplanned reoperations: **a** The types of initial surgery; **b** The incidence of unplanned reoperation following a variety of initial surgery types; **c** The causes of unplanned reoperation(Others: bronchopleural fistula in three cases, torsion or stenosis of right middle bronchus in three cases and pulmonary bulla rupture in one case); **d** The interval time between the initial surgery and the unplanned reoperation; *: Two reoperations were performed in each of three cases

A total of forty-one patients (0.53%) received 44 unplanned reoperations among the 7732 patients. The clinical, demographical, and surgical characteristics are shown in Table 1 and Fig. 1a, respectively.

**Table 1:** Clinical and demographical characteristics of 41 patients who received unplanned reoperations

Characteristics	Classification	*N* (%)
Gender	Male Female	36 (87.8%) 5 (12.2%)
Age(years)	X ± SD	60.1 ± 9.1
Smoker History	Yes No	28 (68.3%) 13 (31.7%)
The usage of platelet aggregation inhibitor	Yes No	3 (7.3%) 38 (92.7%)
Comorbidities^†^	DM Hypertension CHD Cerebral Infarction None	10 (24.4%) 6 (14.6%) 2 (4.9%) 1 (2.4%) 25 (61.0%)
Tumor location	Right upper lobe	17 (38.6%)
	Right middle lobe	2 (4.6%)
	Right lower lobe	11 (25.0%)
	Left upper lobe	7 (15.9%)
	Left lower lobe	6 (13.6%)
	The left hilar	1 (2.3%)
Histology	Benign	9^‡^ (20.5%)
	Malignant	35^§^ (79.5%)
TNM stage	Stage 0 Stage I Stage II Stage III	3 20 10 2
Surgery approach	RATS	10
	VATS	19
	Thoracotomy	12
Lymph node dissection	Yes No	33 8

^†^3 patients had two comorbidities simultaneously

^‡^Four inflammatory pseudotumors and four tuberculoma; 1 patient had bi-primary benign tumor

^§^17 adenocarcinoma, 12 squamous cell carcinoma, one neuroendocrine carcinoma, one small cell lung cancer, one large cell lung cancer and, one poorly differentiated carcinoma; 2 patients had bi-primary lung cancer

*DM* diabetes mellitus; *CHD* coronary heart disease; *VATS* Video-Assisted thoracoscopic surgery; *RATS* Robot-assisted thoracic surgery

Eight patients with benign tumor included four inflammatory pseudotumors and four tuberculomas. Thirty-three patients with malignant tumor included 17 adenocarcinoma, 12 squamous cell carcinoma, one neuroendocrine carcinoma, one small cell lung cancer, one large cell lung cancer, and one poorly differentiated carcinoma. Moreover, bi-primary benign tumor occurred in one case and bi-primary lung cancer happened in two cases. The morbidities of unplanned reoperations in each side seemed to be similar (Left vs. Right: 12/3231 vs. 29/4501, *p* = 0.103).

As shown in Fig. 1a, the initial surgery of these 41 patients included lobectomy, pneumonectomy, wedge resection, pneumonectomy, lobectomy plus segmentectomy, segmentectomy, and exploratory thoracotomy. Intriguingly, in all 7732 cases, the incidence of unplanned reoperation following lobectomy plus segmentectomy was the highest as compared to the other types of surgeries (2/16, 12.5%, Fig. 1b).

As shown in Fig. 1c, the top one complication leading to reoperations was hemothorax, including active hemorrhage (23/44, 52.3%) and clotted hemothorax (6/44, 13.6%). In addition, chylothorax accounted for 18.2% (8/44). The other causes (7/44, 15.9%) included bronchopleural fistula (3/44, 6.8%), torsion or injury of the right middle bronchus (3/44, 6.8%), and pulmonary bulla rupture (1/44, 2.3%).

As shown in Fig. 1d, the interval time between the initial and reoperation were 200–400 h, except for active hemorrhage group (20.7 ± 20.4 h) which required urgent surgical treatment.

**Fig. 2:**
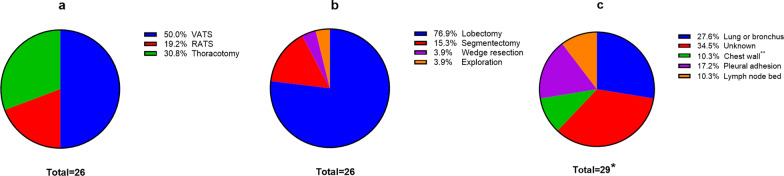
The clinical characteristics of the patients needed unplanned reoperations due to bleeding: **a** The incisions and approaches of the initial surgery; **b** The types of the initial surgery; **c** The bleeding sites leading to hemothorax; *: Two reoperations in each of three cases; **: Bleeding from the surgical incision in one case, bleeding at the drainage tube zone in one case, bleeding of rib stump in one case

Unfortunately, the bleedings resulted in 29 reoperations in 26 patients, including two reoperations in each of three cases. The approaches of the initial surgery of these 26 cases included VATS (*n* = 13)**,** thoracotomy (*n* = 8), and RATS (*n* = 5) (Fig. 2a). Furthermore, these 26 cases initially underwent lobectomy (76.9%), segmentectomy (15.3%), wedge resection (3.9%), and exploratory surgery (3.9%), as shown in Fig. 2b. The bleeding sites are shown in Fig. 2c, including eight (27.6%) from lung or bronchus, ten (34.5%) with the unknown reason (six clotted hemothorax and four active hemorrhages), five (17.2%) from pleural adhesions, three (10.3%) from chest wall vessels, and three (10.3%) in lymph node bed. Therefore, the bleedings sites were divided as Bronchus & lung (*n* = 8) and Others (*n* = 21), as shown in Table 2. Intriguingly, extensive pleural adhesions are more prone to bleedings from pleural adhesion, lymph node bed or chest wall vessels (most bleeding without definite reason can be attributed to chest wall vessels [9, 10]), rather than from Bronchus & lung vessels (*p* = 0.044).

**Table 2:** Clinical and demographical characteristics of patients with hemothorax

Characteristics	Bronchus & lung (*n* = 8^†^)	Others^‡^ (*n* = 21)	t	*p*
Age(years)	66.6 ± 10.3	59.9 ± 9.5	1.677	0.105
*Gender*				1.000
Male	7	18		
Female	1	3		
*Smoking history*				0.675
Yes	5	15		
No	3	6		
*Comorbidities*				1.000
Yes	3	9		
No	5	12		
*PLT aggregation inhibitor*				0.300
Yes	2	2		
No	6	19		
Tumor size(cm)	2.58 ± 1.31	2.89 ± 1.47	− 0.524	0.605
*Pathology diagnosis*				0.647
Benign	1	5		
Malignant	7	16		
*Surgical incision*				1.000
Minimally invasive	5	13		
Thoracotomy	3	8		
*Surgical procedure*^*§*^				0.622
Lobectomy	5	17		
Sub lobectomy	2	4		
Initial operative time(m)	145.6 ± 44.1	163.3 ± 54.8	− 0.815	0.422
*Pleural adhesions*				0.044
Yes	1	12		
No	7	9		
*Kinds of hemothorax*				0.148
Active and progressive	8	15		
Clotted	0	6		
Treatment for bleeding				
*First reoperation*				–
Suture	5	3		
Electrocoagulation	2	6		
Exploration	0	10		
*Second reoperation*^¶^				–
Suture	0	1		
Electrocoagulation	0	1		
Additional Lobectomy	1	0		
Perioperative death	1	3		1.000

^†^The eight bleedings from Bronchus & lung included four at the pulmonary vascular stump, two from rupture of stapled margin due to the thickness and tension of pulmonary tissues, one continuous exudation of parenchyma, and one at the bronchial artery

^‡^Including bleeding from the chest wall, pleural adhesion, lymph node bed, and unknown reason

^§^Exclude a patient with exploratory thoracotomy, and combine wedge resection and segmentectomy into Sub lobectomy

^¶^Suture was performed for hemostasia in the first reoperation

*PLT* platelet

Suture or electrocoagulation was performed to stop bleedings in case of definite sites. And clearance of pleural cavity was administrated if the bleeding site can’t be identified. Moreover, an additional right middle lobectomy was performed in one patient with initial lower lobectomy, because only manipulation by suture can't stop the bleeding at the pulmonary artery trunk.

Chylothorax (Table 3) occurred in seven patients with right upper or lower lobectomy, and one with left pneumonectomy plus lymphadenectomy. The reoperation for right chylothorax was conducted for en bloc ligation of the thoracic duct and peripheral tissues located 5 cm above diaphragmatic hiatus, and the reoperation for left chylothorax was ligation of the thoracic duct in the aortopulmonary window plus 5 cm above the diaphragmatic hiatus.

**Table 3:** The clinical information of eight cases with chylothorax

Cases	Pathological diagnosis	Initial surgery	Drainage volume (mL/d)	Interval time between initial and second operation(d)
Case 1	AC	RATS RUL plus lymphadenectomy	1426	5.0
Case 2	AC	RATS RUL plus lymphadenectomy	380	9.0
Case 3	AC	RATS RUL plus lymphadenectomy	1062	2.8
Case 4	SCC	RATS RLL plus lymphadenectomy	381	13.7
Case 5	SCC	Th LTP plus lymphadenectomy	1560	18
Case 6	LCC	VATS RLL plus lymphadenectomy	633	12.8
Case 7	SCC	VATS RLL plus lymphadenectomy	2163	6.4
Case 8	AC	RATS RUL plus lymphadenectomy	1062	2.8

*RATS* Robotic-assisted thoracic surgery; *VAST* Video-assisted thoracic surgery; *Th* thoracotomy; *RUL* right upper lobe; *RLL* right lower lobe; *LTP* left total pneumonectomy; *AC* Adenocarcinoma; *SCC* Squamous cell carcinoma; *LCC* Large cell carcinoma

The bronchopleural fistula occurred in three lung cancer patients, following left pneumonectomy in two cases and right lower lobectomy plus RS1 segmentectomy in one case (fistula at the stump of RS1).

The left upper pulmonary bulla ruptured in one patient who suffered from inflammatory pseudotumor following left lower lobectomy, leading to the reoperation to resect bulla.

The torsion or stenosis of the right middle bronchus happened in three cases. Right upper lobectomy or right upper lobectomy plus RS6 segmentectomy caused right middle bronchus torsion in two patients who were diagnosed with tuberculoma and lung cancer each. Both of them received anatomical restoration and fixation of the residual pulmonary. In the third patient with lung cancer, right lower lobectomy caused the postoperative stenosis of the right middle bronchus because the bronchus can’t be isolated from calcified hilar lymph nodes and had to be transected simultaneously with these lymph nodes by a stapler. Thereafter, reoperation to resect the right middle lobe was performed after the middle lobe atelectasis was confirmed by postoperative chest X-ray and stenosis of right middle bronchus was defined by bronchoscopy.

. Among the 41 patients, 34 patients were discharged smoothly, whereas seven cases suffered from more intractable complications following unplanned reoperation, including two ARDS, two cardiopulmonary failure, one pyothorax, one prolonged air leakage, and one severe pneumonia. Unfortunately, among the 41 patients, there were five deaths (12.2%), as shown in Table 4.

**Table 4:** The clinical information of five death cases

Cases	Age (Y)	Comorbidities	Initial surgery	Pathological diagnosis	Reason for reoperation	Reoperation	Cause of death
#1	76	No	RATS RLL lobectomy	AC	RML Atelectasis	RML lobectomy	Cardiopulmonary failure
#2	79	Cerebral Infarction^†^	RLL lobectomy	AC	Active bleeding in artery stump	Exploration	ARDS
					Re-bleeding in artery stump	RML lobectomy	
#3	71	Hypertension, CHD^†^	RUL lobectomy	PDC	Clotted hemothorax due to pleural bleeding	Exploration	Severe pneumonia
#4	76	Hypertension, CHD^†^	VATS LLL lobectomy	SCC	Clotted hemothorax without Definite bleeding site	Exploration	Cardiopulmonary failure
#5	49	DM	LUL lobectomy plus the second costatectomy	AC	clotted hemothorax without Definite bleeding site	Exploration	ARDS

^†^Treatment with platelet aggregation inhibitor, replaced with low molecular weight heparin one week before surgery

*DM* diabetes mellitus; *CHD* coronary heart disease; *AC* Adenocarcinoma; *PDC* poorly differentiated carcinoma; *SCC* Squamous cell carcinoma

## Discussion

The incidence of unplanned reoperation is 0.53% in our study, which is higher than the published 0.27% (after lung cancer surgeries) [8], probably due to the included benign tumors herein. Indeed, the benign tumors were reported to be more risky to postoperative bleedings than the malignant tumor, potentially due to inflammation [11]. Among the initial surgeries caused reoperations, lobectomy plus segmentectomy is the most risky probably due to more complicated manipulations and more bronchus stumps.

In our study focusing on unplanned reoperation, postoperative bleeding is the top one reason and the mean interval time between the first and second operation was less than 24 h due to the urgent treatment for active and progressive hemothorax. Among the six patients with clotted hemothorax, two patients were readmitted for reoperation after discharge. One was discharged after the removal of continuous suction drainage due to prolonged air leakage (seventeen days following left upper lobectomy plus lymphadenectomy). Unfortunately, the patient was readmitted to our hospital sixteen days later due to dyspnea, and the chest CT confirmed clotted hemothorax and the destroyed left lower lobe probably due to secondary infection and atelectasis. Thereafter, the patient underwent clearance of pleural cavity and left lower lobectomy. The other one was discharged after the removal of seven-day-drainage for pleural effusion (eight days followingright lower lobectomy). However, the patient was readmitted after six days, due to dyspnea and right hydropneumothorax confirmed by chest X-ray, and the exploration and clearance of the pleural cavity were performed emergently. Fortunately, the two patients recovered and rehabilitated smoothly. Therefore, chest X-ray film in two weeks after discharge is critical, especially for patients with newly developed dyspnea. Additionally, the expansion of lung is critical to prevent hemothorax. The intraoperative residual lung expansion should be proven to have no major air leak detected, and repaired if detected. In the postoperative period, early mobilization and pulmonary rehabilitation increase lung expansion and limit the formation of hemothorax.

The bleeding sites from the lung or bronchus can locate at the vascular stump, the bronchial stump with bronchial artery, and the surgical edges of pulmonary parenchyma. Herein, the two cases suffered from bleedings from the stapled surgical edges of pulmonary parenchyma due to inappropriate chosen of staple cartridge mismatching the thickness of tissues, including one rupture of thick tissues and one closure failure of thin tissues. Therefore, pulmonary parenchyma should be evaluated prior to the selection of a suitable and matched staple cartridge. Interestingly, one-third of these 31 hemothorax (10 cases) including 5 clotted hemothorax and 5 active hemorrhages, had no definite bleeding sites, accordant to the reported incidence (37.7–41%) [8, 9], which can be attributed to chest wall vessels and may be ignored during reoperation via the initial incision [10]. Furthermore, our study indicated pleural adhesion is also a risk factor for the bleedings from the chest wall and pleural, especially in some “blind” areas, e.g., vicinity of incision and costophrenic angle. However, due to the limited field of the view, some areas may be overlooked in minimal invasive approach with the use of small incision [12].

Normally, suture or electrocoagulation is effective to stop bleedings except for three cases who unfortunately underwent the second reoperation for re-hemorrhage. In the first patient with long-term administration of antiplatelet drugs for cerebral infarction before the initial right lower lobectomy, the re-operation was performed to suture pulmonary artery stumps, however, the re-bleeding at the interlobar artery caused the third operation to remove right middle lobectomy. In the second patient, reoperation was performed to suture and stop bleedings at the stump of the right inferior pulmonary vein, however, the bleeding from thoracic cavity adhesion caused the third operation. In the third patient, the bleedings from the surgical edges of pulmonary parenchyma and pleural cavity adhesion caused the second and third reoperation, respectively. Hence, we should treat the vascular stump carefully with the reinforced suture and detect the pleural cavity meticulously during the second operation.

Incidence of chylothorax after pulmonary resection was reported to be 0.7–2.3% [13, 14]. Conservative therapies [14] in case of the small amount of chyle may be effective, including chemical pleurodesis [15] and percutaneous catheter embolization of the thoracic duct [16]. About 10% of chylothorax after lobectomy without response to conservative therapies required reoperation for thoracic duct ligation [17], while the chylothorax occurring after pneumonectomy usually required reoperation [18]. The incidence of chylothorax was remarkably increased in pathological N2 disease which requires more radical mediastinal lymph node dissection [17]. The prophylactic ligation of the thoracic duct branch is recommended in the patients who underwent right lobectomy and 4R lymph-node resection [19]. In our study, among all the 7732 patients, the incidence of chylothorax requiring reoperation is 0.1%, accounting for the top two reasons for reoperation. Therefore, it is essential to carefully protect the thoracic duct and main lymphatic vessel during operation, and prophylactic ligation of the thoracic duct is necessary in cases of suspected oily-like exudation.

The incidence of lobar torsion following pulmonary resections is relatively rare (0.089%–0.3%) [20, 21], potentially leading to the extended lobectomy and even pneumonectomy. Torsion of the right middle lobe is most frequently, especially following right upper lobectomy [22]. Staple or suture the middle lobe to the adjacent lobe or chest wall is recommended to prevent lobe torsion, especially for the patients with complete and thin pulmonary fissure [20, 21].

The bronchopleural fistula is a common complication after lobectomy, segmentectomy, or pneumonectomy (ranging from 4.5 to 20% after pneumonectomy) [23]. In our study, there were three cases suffered from bronchopleural fistula (3/44, 6.8%). Among them, two initially underwent left pneumonectomy and subsequently underwent the reoperation to suture the fistula stump after six days and ten days each. One case initially underwent right lower lobectomy plus RS1 segmentectomy, however, empyema was found after 16 days due to fistula at B1 stump, which was sutured urgently. Postoperative air leak discontinued after five months treatment including anti-inflammation and nutritional supplementation. We acknowledge that thoracoplasty to remove the matching ribs, rather than suture the bronchial stump, may be more appropriate for this case.

## Conclusions

This study analyzed the common reasons for the unplanned reoperations after pulmonary tumor resection and provided the pivotal points which should be focused onduring the initial surgery to prevent the postoperative crises. Firstly, bronchi or vessel stumps, the surgical edges of lung parenchyma, and pleural adhesions should be checked carefully prior to closing incision, especially in those patients with long-term administration of antiplatelet drugs. Secondly, prophylactic ligation of the thoracic duct should be recommended in case of the suspected oily-like exudation in the lymph node bed, especially for the thoracic surgery on the right side or left pneumonectomy. Thirdly, smooth expansion of the middle lobe is important to avoid narrowing and torsion prior to the introduction of stapler and transection of bronchus, especially with the calcified paratracheal lymph nodes.

## Data Availability

The datasets used and/or analyzed during the current study are available from the corresponding author on reasonable request.

## Abbreviations

VATS: Video-Assisted thoracoscopic surgery
RATS: Robot-assisted thoracic surgery
DM: Diabetes mellitus
CHD: Coronary heart disease
PLT: Platelet
Th: Thoracotomy
RUL: Right upper lobe
RLL: Right lower lobe
LTP: Left total pneumonectomy
AC: Adenocarcinoma
SCC: Squamous cell carcinoma
LCC: Large cell carcinoma
PDC: Poorly differentiated carcinoma
SCC: Squamous cell carcinoma

## References

[1] Cho JH (2021). Establishment of minimally invasive thoracic surgery program. J Chest Surg.

[2] Napolitano MA, Sparks AD, Werba G, Rosenfeld ES, Antevil JL, Trachiotis GD. Video-assisted thoracoscopic surgery lung resection in United States veterans: trends and outcomes versus thoracotomy. In: The Thoracic and cardiovascular surgeon; 2021. 10.1055/s-0041-1728707 34044463

[3] Kent MS, Hartwig MG, Vallieres E, Abbas AE, Cerfolio RJ, Dylewski MR et al. Pulmonary open, robotic and thoracoscopic lobectomy (PORTaL) study: an analysis of 5,721 cases. Ann Surg; 2021. 10.1097/SLA.0000000000005115 PMC9891268 34534988

[4] Zhang Y, Chen C, Hu J, Han Y, Huang M, Xiang J (2020). Early outcomes of robotic versus thoracoscopic segmentectomy for early-stage lung cancer: a multi-institutional propensity score-matched analysis. J Thorac Cardiovasc Surg.

[5] Dell'Amore A, Lomangino I, Cannone G, Terzi S, Pangoni A, Lorenzoni G et al. Comparison of operative and postoperative characteristics and outcomes between thoracoscopic segmentectomy and lobectomy for non-small-cell lung cancer: a propensity score matching study from the Italian VATS Group Registry. Eur J Cardio-thoracic Surg Off J Eur Assoc Cardio-thoracic Surg; 2021. 10.1093/ejcts/ezab430 34643695

[6] Lightner AL, Glasgow AE, Habermann EB, Cima RR (2017). Returns to operating room after colon and rectal surgery in a tertiary care academic medical center: a valid measure of surgical quality?. J Gastrointest Surg.

[7] Louie BE, Wilson JL, Kim S, Cerfolio RJ, Park BJ, Farivar AS (2016). Comparison of video-assisted thoracoscopic surgery and robotic approaches for clinical stage I and stage II non-small cell lung cancer using the society of thoracic surgeons database. Ann Thorac Surg.

[8] Li J, Xue Q, Gao Y, Mao Y, Zhao J, Gao S (2020). Bleeding is the most common cause of unplanned return to operating room after lung cancer surgeries. J Thorac Dis.

[9] Sirbu H, Busch T, Aleksic I, Lotfi S, Ruschewski W, Dalichau H (1999). Chest re-exploration for complications after lung surgery. Thorac Cardiovasc Surg.

[10] Solaini L, Prusciano F, Carletti M (2011). Video-assisted thoracoscopic surgery for postoperative hemothorax. Thorac Cardiovasc Surg.

[11] Udelsman BV, Soni M, Madariaga ML, Fintelmann FJ, Best TD, Li SS (2020). Incidence, aetiology and outcomes of major postoperative haemorrhage after pulmonary lobectomy. Eur J Cardio-thoracic Surg Off J Eur Assoc Cardio-thoracic Surg.

[12] Sezen CB, Bilen S, Kalafat CE (2019). Unexpected conversion to thoracotomy during thoracoscopic lobectomy: a single-center analysis. Gen Thorac Cardiovasc Surg.

[13] Kutlu CA, Sayar A, Olgac G, Akin H, Olcmen A, Bedirhan MA (2003). Chylothorax: a complication following lung resection in patients with NSCLC–chylothorax following lung resection. Thorac Cardiovasc Surg.

[14] Takuwa T, Yoshida J, Ono S, Hishida T, Nishimura M, Aokage K (2013). Low-fat diet management strategy for chylothorax after pulmonary resection and lymph node dissection for primary lung cancer. J Thorac Cardiovasc Surg.

[15] Zhang K, Li C, Zhang M, Li Y (2021). Treatment of Chylothorax complicating pulmonary resection with hypertonic glucose Pleurodesis. J Cardiothorac Surg.

[16] Ishida H, Nakazawa K, Yanagihara A, Umesaki T, Taguchi R, Yamada A et al. Chylothorax associated with lymphatic reflux in a thoracic duct tributary after lung cancer surgery. Thorac Cancer; 2021. 10.1111/1759-7714.14062 PMC8327699 34152082

[17] Bryant AS, Minnich DJ, Wei B, Cerfolio RJ (2014). The incidence and management of postoperative chylothorax after pulmonary resection and thoracic mediastinal lymph node dissection. Ann Thorac Surg.

[18] Le Pimpec-Barthes F, D'Attellis N, Dujon A, Legman P, Riquet M (2002). Chylothorax complicating pulmonary resection. Ann Thorac Surg.

[19] Liu Z, Du M, Liang Y, Ju S, Li X, Gao Y (2020). Prophylactic ligation of the thoracic duct branch prevents chylothorax after pulmonary resection for right lung cancer. Surg Today.

[20] Cable DG, Deschamps C, Allen MS, Miller DL, Nichols FC, Trastek VF (2001). Lobar torsion after pulmonary resection: presentation and outcome. J Thorac Cardiovasc Surg.

[21] Taira N, Kawasaki H, Takahara S, Furugen T, Atsumi E, Ichi T (2018). Postoperative lung torsion with retained viability: the presentation and surgical indications. Heart Lung Circ.

[22] Ziarnik E, Grogan EL (2015). Postlobectomy early complications. Thorac Surg Clin.

[23] Alpert JB, Godoy MC, Degroot PM, Truong MT, Ko JP (2014). Imaging the post-thoracotomy patient: anatomic changes and postoperative complications. Radiol Clin North Am.

